# Topography Measurement of Large-Range Microstructures through Advanced Fourier-Transform Method and Phase Stitching in Scanning Broadband Light Interferometry

**DOI:** 10.3390/mi8110319

**Published:** 2017-10-26

**Authors:** Yi Zhou, Yan Tang, Yong Yang, Song Hu

**Affiliations:** 1 State Key Laboratory of Optical Technologies for Microfabrication, Institute of Optics and Electronics, Chinese Academy of Sciences, Chengdu 610209, China; alanzhouyi@163.com (Y.Z.); yangyong@ioe.ac.cn (Y.Y.); husong@ioe.ac.cn (S.H.); 2 University of Chinese Academy of Sciences, Beijing 100049, China

**Keywords:** spatial modulation, dimensional metrology, phase stitching, microstructure

## Abstract

Scanning broadband light interferometry (SBLI) has been widely utilized in surface metrology due to its non-contact and high-accuracy method. In SBLI, phase evaluation through Fourier Transform (FT) is a prevalent and efficient technique, where the topography measurement can often be achieved through one interferogram. Nevertheless, the accuracy of the FT method would be significantly influenced by intensity modulation depth: “the lower the modulation of the pixel, the higher the error probability of its phase assignment”. If the structure has a large enough range along the *z*-axis, several areas in an individual interferogram would be weakly modulated due to the limited depth of focus (DOF). In this paper, we propose an advanced FT-based method when it comes to large-height structures. Spatial modulation depth is first calculated for each interferogram independently. After that, a binary control mask is reasonably constructed to identify the pixels that are valid for phase unwrapping. Then, a phase stitching method along the *z*-axis is carried out to conduct the large-height topography measurement within a giving field of view. The theoretical principle, simulation, and experimental validation are elaborated to demonstrate that the method can achieve an improved robustness for the reconstruction of large-range microstructures, the advantages of which include the elimination of stepping errors, the suppression of light fluctuations, and the freedom of a limited DOF.

## 1. Introduction

Three-dimensional measurement of microstructure is an area with growing needs and interest since it plays an important role in addressing quality issues and observing the performance of micro products. Given its high-precision, non-contact, and full-field method, the “topography measurement,” which is based on scanning broadband light interferometry (SBLI), has been widely applied in measuring and calibrating microstructures ranging from the nanometer to the micrometer scale [1,2,3,4]. Nonetheless, the accuracy of most previously recovering algorithms, such as the centroid algorithm, the peak location method, and the envelope detection technique, are at times negatively influenced by moving errors and intensity fluctuations [5,6,7,8,9]. Typically, phase evaluation is a general and effective method for precise metrology in SBLI. In phase measuring profilometry, there are mainly two techniques to retrieve the phase data including phase-shifting interferometry (PSI) and Fourier transform (FT) in the spatial frequency domain. For PSI, the phase mapping from *N* frames to obtain a wrapped phase is pixel-to-pixel analysis. Nevertheless, the PSI-based method would be significantly limited in a complex environment, since the accuracy strongly depends on stepping precision and light stability [10,11,12,13,14,15]. 

Contrarily, the Fourier transform is always a reliable technique to retrieve the phase distribution without moving errors and light fluctuations, for the reason that every interferogram is analyzed independently and filtering is adopted in the spatial frequency domain [16,17,18,19]. Additionally, as was pointed out in [20], “to make reliable phase measurements, the incident intensity must modulate sufficiently at each detector point to yield an accurate phase.” In the FT method, the accuracy of phase measurement directly depends on the modulation depth, “the lower the modulation depth of the pixel, the higher the error probability (the possibility to cause errors) of its phase assignment” [21,22,23]. Actually, when the measurement sample has a large enough range along the *z*-axis, several parts in the interferogram would be weakly modulated due to the limited depth of focus (DOF) of the imaging system. Particularly, devices in many science domains, including biomedical engineering, chemistry, and integrated circuits (ICs), are at times large-height structures [24,25,26,27]. In that case, when the height range is beyond the DOF, only a part of areas can be recognized at a definite scanning position, causing difficulty to reshape the topography within a giving field of view through one interferogram. 

In this paper, we propose an advanced FT-based method and phase stitching technique when it comes to large-height structures. Spatial modulation depth is first calculated in the {*xy*} plane (imaging plane) for each charge-coupled device (CCD) image independently. After that, a binary control mask, which is constructed based on modulation threshold, is developed to identify the pixels that are valid for phase unwrapping in each interferogram. The threshold limit of the intensity modulation is appropriately set, and all pixels that do not modulate sufficiently are excluded. Then, a phase stitching method along the *z*-axis is proposed to complete the large-range measurement. The principle has been elaborated in detail, which contains modulation extraction, identification for valid pixels, the phase stitching process, and reconstruction algorithms. Simulation on a slope plane is organized to explain the procedure and verify the effectiveness. Additionally, the experimental results on a micro lens with a height of nearly 3000 nm show that the proposed method can precisely reshape the surface topography, the advantages of which include freedom of stepping errors, the suppression of light fluctuations, and a significant improvement in the measurement range.

## 2. Principle 

**Figure 1:**
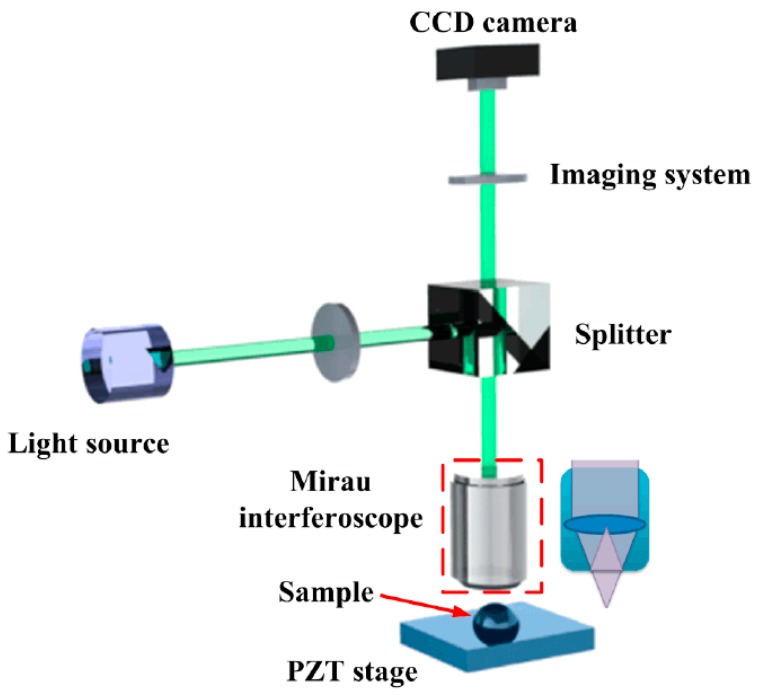
Schematic of Mirau correlation microscope-based scanning broadband light interferometry (SBLI) system.

Figure 1 illustrates a Mirau correlation microscope-based system of SBLI for dimensional metrology of micro samples. In this system, a broadband light is utilized to illuminate the test and reference surface. Light transmits through the interferometry objective, which contains a beam splitter and a several-millimeter reference mirror, where the light is divided into two beams by the beam splitter (the transmittance ratio is about 50%). One is reflected on the reference mirror and the other is on the measurement structure. The superposition beams are relayed to the CCD camera and produce an interferogram of the sample topography that can be captured by the individual CCD pixels. This technique is achieved by vertically scanning the sample in a high-precision piezo-electric transducer (PZT) stage, where the interferograms can be rapidly detected at different scanning positions. 

In broadband light interferometry, when intensities from the measurement arm and reference arm are equal, the interference intensity field can be described as

(1) $$I\left(z\right)=I_{0}\left\{1+\exp\left[-{\left(\frac{z-h-z_{0}}{l_{c}}\right)}^{2}\right]\cos\left(4\pi \frac{z-h-z_{0}}{\lambda_{0}}\right)\right\}$$

where *I*_0_ is the background irradiance, *z* presents the scanning distance, *h* describes the sample height, *z*_0_ denotes the length of reference arm, *l_c_* illustrates the coherence length of the light source, and *λ*_0_ is the central wavelength [5,28]. When an interferogram is obtained at a definite scanning position *z_n_*, the irradiance distribution can be expressed as

(2) $$I(x,y)=A(x,y)+B(x,y)\cos\left(4\pi \frac{z_{n}-h(x,y)-z_{0}}{\lambda_{0}}\right),\;\mathrm{with}\;B(x,y)=A(x,y)\exp\left[-{\left(\frac{z_{n}-h(x,y)-z_{0}}{l_{c}}\right)}^{2}\right]$$

where *A*(*x*, *y*) presents the background intensity in a definite position, *B*(*x*, *y*) can be deemed as the fringe contrast. 

In this method, a measured sample is slightly tilted to develop a small angle between the light axis and sample surface to produce a carrier frequency, which gives

(3) $$I(x,y)=A(x,y)+B(x,y)\cos\left(4\pi \frac{z_{n}-(h(x,y)-kx)-z_{0}}{\lambda_{0}}\right)$$

where kx is the initiatively tilted slope along the *x* axis, while k is the slope factor. Then, the equation can be rewritten as

(4) $$I(x,y)=A(x,y)+B(x,y)\cos\left(4\pi \frac{kx}{\lambda_{0}}+4\pi \frac{z_{n}-z_{0}-h(x,y)}{\lambda_{0}}\right)$$

To simplify the expression, the intensity distribution can be illustrated as

(5) $$I(x,y)=A(x,y)+B(x,y)\cos\left(2\pi f_{0}x+\phi (x,y)\right)$$

where $\phi (x,y)=4\pi \frac{z_{n}-z_{0}-h(x,y)}{\lambda_{0}}$, $f_{0}=2\frac{k}{\lambda_{0}}$.

For extracting the sideband signal in the frequency domain, Equation (5) can also be rewritten in the following form according to Euler’s formula,

(6) $$I(x,y)=A(x,y)+C(x,y)e^{i2\pi f_{0}x}+C{\left(x,y\right)}^{*}e^{-i2\pi f_{0}x},\;\mathrm{with}\;C(x,y)=\frac{1}{2}B(x,y)e^{-i\phi (x,y)}$$

where ${}^{*}$ presents the complex conjugate. Then, we apply two-dimensional Fourier transform of Equation (6) to obtain the frequency spectra, as expressed in

(7) $$G\;(f_{x},f_{y})=G_{A}(f_{x},f_{y})+D_{-1}(f_{x},f_{y})⊗G_{-1}(f_{x},f_{y})+D_{1}(f_{x},f_{y})⊗G_{1}(f_{x},f_{y})),\;\mathrm{with}\;D_{-1}(f_{x},f_{y})=F[C(x,y)]$$

where *G*(*f_x_*, *f_y_*) denotes the Fourier spectra, *G_A_*(*f_x_*, *f_y_*) is the zero frequency, *G_−1_*(*f_x_*, *f_y_*) and *G_1_*(*f_x_*, *f_y_*) are the sideband signals, ⊗ is the convolution symbol, and F represents the 2D Fourier transform. We extract either of the two spectra on the carrier, say *D_−1_*(*f_x_*, *f_y_*), and apply inverse Fourier transform to obtain *C*(*x*, *y*) Then, the spatial modulation depth and phase distribution in the {*xy*} plane can be calculated as

(8) M(x,y)=|B(x,y)|=2|C(x,y)|

(9) ϕ(x,y)=Angle[C(x,y)]

where *M*(*x*, *y*) is expressed as the spatial modulation depth, ‘Angle’ is the function to obtain the wrapped phase values within (−π, π). After that, unwrapped phase algorithms are utilized to obtain the continuous phase values, through which the surface topography can be effectively reshaped. To guarantee the accuracy of the measurement, the pixels with lower modulation should be removed from consideration in phase unwrapping. Commonly, when the measurement sample has a large enough range along the *z*-axis, the intensity modulation depth will decrease significantly due to the limited DOF. Therefore, an estimation of modulation depth is essential to satisfactorily retrieve the phase assignment. 

Initially, two-dimensional Fourier transform and signal processing of individual interferogram are conducted to calculate both intensity modulation depth and discrete phase. Then, an effective threshold limit is reasonably determined for distinguishing the pixels that are valid and invalid for phase unwrapping, where a binary mask is logically constructed (the valid pixels are set to be one, and invalid ones are generated as zero). If the threshold is set too high, excluded areas will be enlarged. In that case, although the obtained phase will have higher reliability, it will take more time and cause fewer intersections between adjacent frames. On the contrary, if the threshold is established too low, the phase unwrapping procedure will lack reliability. 

In this technique, we utilize the Diamond phase-unwrapping algorithm to calculate continuous phase, as described in [29]. The algorithm firstly identifies a seed point, which will spread to four points nearby. After that, the four points will serve as the second group of seed points. These seed points will spread again to four points nearby the second group in turn and pass through all of the effective points by a diamond path. Seed points will eventually bring about the phase unwrapping in the whole image. In the method, the sample is scanned by the PZT scanner along the *z*-axis, where a sequence of interferograms will be obtained at different scanning positions. When the step pace is set appropriately, a number of pixels will be valid in both adjacent interferograms. In that case, the unwrapped phase of the valid areas in two frames can be utilized for phase stitching process along the *z*-axis.

## 3. Simulation

**Figure 2:**
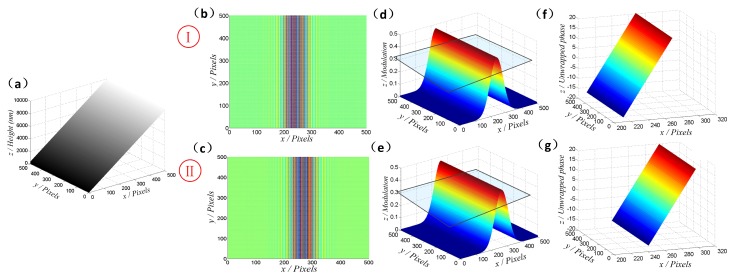
Process for continuous phase calculation through the modulation identification. (**a**) The simulated large-height slope. (**b**,**c**) Fringe patterns at two different scanning positions. (**d**,**e**) The corresponding spatial modulation of the obtained interferograms, with quadrangle indicating the threshold limit. (**f**,**g**) The continuous phase values only for valid pixels.

To explain the proposed procedure specifically, the simulation has been conducted on a large-range slope. The height difference of the slope sample ranges greatly along the *z*-axis, as shown in Figure 2a. In the process, the central wavelength of simulated broadband light source (enveloped by Gaussian curve, full width at half maximum (FWHM) is 200 nm) is 580 nm. Additionally, a sequence of interferograms will be captured in the scanning direction, from which we select two adjacent frames as the examples. In the interference frames, it can be noted that some areas are of a high fringe-pattern contrast, while others are lowly modulated, as illustrated in Figure 2b,c. Then, 2D Fourier transform and signal processing methods are applied to obtain the spatial modulation depth in the {*xy*} plane for each frame, as shown in Figure 2d,e. After that, a binary mask will be generated referring to the valid pixels, whose modulation depth is above the threshold limit (the quadrangle denotes the threshold). Consequently, absolute phase values will be calculated only for the valid pixels in each interferogram, as presented in Figure 2f,g.

**Figure 3:**
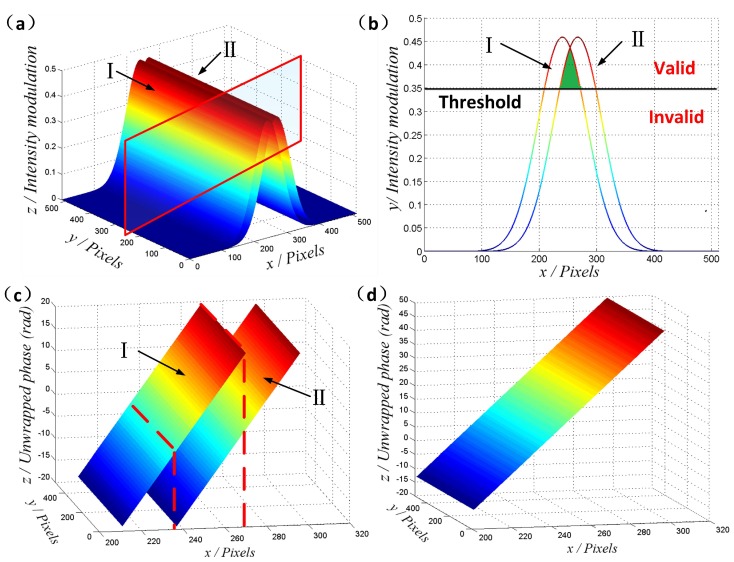
An example showing the steps of the phase stitching method. (**a**) The spatial modulation of two adjacent interferograms. (**b**) Cross section at the marked position in (**a**) and the identification of valid pixels. (**c**) The phase stitching process through intersection areas, with a red line indicating the different values of the same areas. (**d**) Absolute phase distribution through the stitching process.

Figure 3a shows the modulation distribution of two adjacent interferograms, illustrating the trend of fringe-pattern contrast. Additionally, with the purpose of making it clear to explain the stitching method, a cross section indicated by a rectangle is presented in Figure 3b, where there are two modulation curves, expressed as I and II. The green section denotes the intersection of both curves, which means that these areas are commonly valid in both interferograms. Note that the phase values of intersection pixels are calculated in both adjacent frames, but the values are not same because of the different scanning positions, as presented in Figure 3c. In the stitching step, the mean values of the intersection areas are first calculated. After that, the difference can be obtained as described in

(10) DV=Mean(Ι)−Mean(ΙΙ)

in which Mean(I) and Mean(II) illustrate the mean values of the intersection in Frames I and II, respectively. Lastly, in order to eliminate the phase difference between the two adjacent frames, the *DV* is added to the phase values of Frame II and the stitched continuous phase is obtained. In that case, the phase distribution of the valid areas in both two interferograms will be calculated, as shown in Figure 3d. It should also be noted that additional frames are required to completely recover the large-height surface topography.

## 4. Experimental Results

An experiment on a large-range micro-dome silica structure (micro lens) is carried out to demonstrate the advanced FT technique and the phase stitching method in SBLI. The experiment setup is developed referring to Figure 1. The system mainly contains a broadband light source (LED illuminant enveloped by Gaussian function whose central wavelength is 560 nm and whose bandwidth is 160 nm), a CCD camera that contains 768 × 576 pixels with a resolution of 6.25 × 6.5 μm, a Mirau interferoscope (50×, NA = 0.55, DOF = 900 nm) produced by the Nikon Company (Tokyo, Japan), a PZT scanning stage (with a resolution of 1.0 nm and a scanning range of 20.0 μm, produced by PI Ceramic Co., Lederhose, Germany), and self-designed imaging components. 

**Figure 4:**
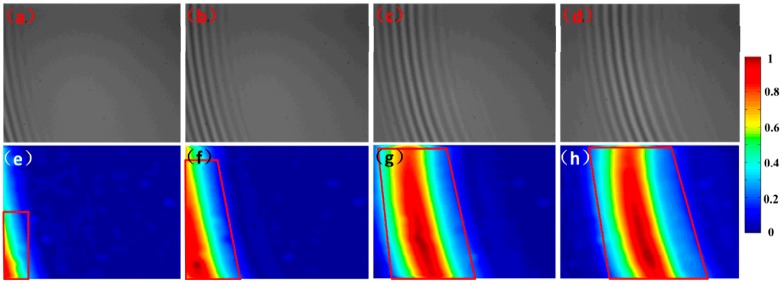
Identification based on spatial modulation. (**a**–**d**) The captured interference frames at different scanning positions. (**e**–**h**) Spatial modulation depth distributions with the red line indicating the satisfactory areas (whose modulation is higher than 0.3).

In the experiment, nine interferograms are used to reshape the topography of the micro lens. Figure 4a–d shows four of the interferograms, which are captured with a step pace of 200 nm. Then, the spatial modulation depth distributions in the {*xy*} plane are obtained for each interferogram independently and the modulation threshold limit is reasonably set according to different materials (with different reflection index) of samples. In order to balance the measurement accuracy and time, the threshold will be set higher if the reflection ability is strong enough; otherwise, the threshold will be adjusted lower. In the latter case, we will try different limit values for improved experimental results. Once the threshold limit is confirmed, the absolute phase values of the valid pixels will be calculated via the FT method, as illustrated in Figure 4e–h. It should be noted that the quadrilateral in the figure is just an example of the indication line, and the boundaries of “satisfactory areas” could actually be confirmed by any other curve. 

**Figure 5:**
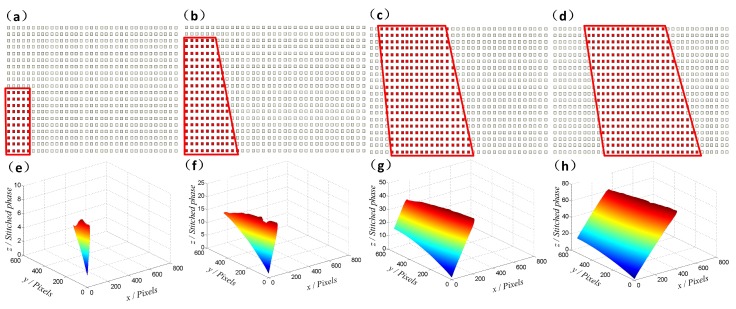
Phase stitching through valid areas. (**a**–**d**) The valid pixels are indicated by the red color, while the invalid ones are blank. (**e**–**h**) The stitched continuous phase obtained frame by frame.

Generally, only some of the pixels in the micro lens are valid in one interferogram due to the limited DOF. Therefore, a stitching process along the *z*-axis is required to calculate the absolute phase of the given field of view. Based on the identification of the modulation threshold, the valid pixels would be successfully recognized, as shown in Figure 5a–d. The red color illustrates the valid pixels, of which the continuous phase will be calculated, while the blank shows the invalid ones. Consequently, the continuous phase can be stitched frame by frame like a growing tree, as shown in Figure 5e–h. 

**Figure 6:**
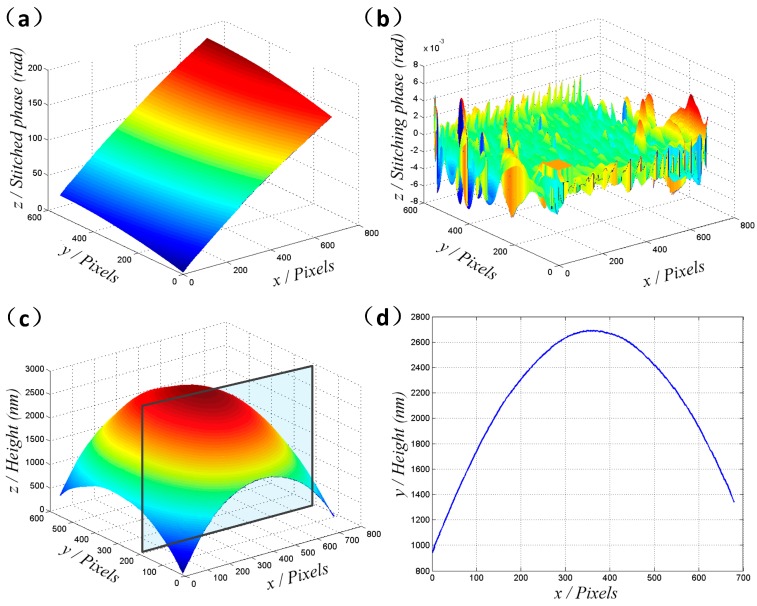
Experimental results and stitching errors. (**a**) The stitched phase values through the nine interferograms. (**b**) Stitching errors. (**c**) The reshaped topography of the micro-dome structure when redundant slope has been removed. (**d**) Cross-section profile at the marked position (*y* = 200) in (**c**).

In the procedure, when all the interferograms are analyzed in sequence, the absolute phase distribution of the sample surface will be obtained, as illustrated in Figure 6a. Since the technique uses mean values of the intersection areas to carry out the stitching method, the phase of individual pixels will be slightly deviated. Here, the stitching errors are defined as

(11) Errors=Phase(ΙΙ)−Phase(Ι)

where Phase(I) are the phase values of the intersection pixels in frame I, and Phase(II) are the phase distributions after adding the *DV* to Frame II of the intersection areas. Therefore, there will be stitching errors for each independent pixel, which are the main causes of measurement errors in the proposed method. It can be seen from Figure 6b that the stitching phase errors are within the range of −0.008–0.008 rad. Accordingly, when it comes to the topography information, Equation (12) will be applied in the Mirau interference system.

(12) $$h(x,y)=\frac{\phi (x,y)}{2\pi }*\frac{\lambda_{0}}{2}$$

From which, the stitching errors of this technique are obtained to be ±0.36 nm.

In addition, a 3D plane-fitting algorithm is applied to obtain the tilting slope in the experiment according to

(13) F(x,y,z)=Ax+By+Cz+D

where *F*(*x*, *y*, *z*) indicates the tilting slope, while the coefficients *A*, *B*, *C*, and *D* can be calculated via the least square method. When the redundantly tilting slope has been removed, the surface topography of the large-height micro-dome structure can be obtained, as presented in Figure 6c,d. In the experiment, we also conducted four repetitions under the same experiment environment, where the results of the positions (*x* = 300 pixels, *y* = 200 pixels and *x* = 400 pixels, *y* = 100 pixels) were obtained, as shown in Table 1. Additionally, in order to demonstrate the accuracy of this method, the results measured by a commercial 3D profiler (New View 8000, Zygo Corporation, Middlefield, CT, USA) are tabulated in Table 1. The micro-dome lens, which is produced by photolithography method, is aspherical and asymmetrical in the surface, so it is feasible to match the topography in SBLI with the results of the 3D profiler through the aspherical shape. Additionally, since the CCD camera used in SBLI is the same as the one applied in the 3D profiler, we can compare the data pixel to pixel to verify the height information. 

As a result, the measurement repeatability can be estimated to be within the range of 0.1%. Meanwhile, the mean errors between the proposed method and the 3D profiler are experimentally obtained to be less than 1.5 nm. We also compare the height information of various other pixels to demonstrate that the stitching errors are almost uniform at all lateral positions, for the reason that the stitching deviations are nearly unvarying for the giving surface. It can also be noted that the height difference of the sample is nearly 3000 nm, which is significantly larger than the DOF of the experiment system. Additionally, the light fluctuations and environmental disturbances can be suppressed in some extent, because the filtering process is adopted in the spatial frequency domain and the background intensity is removed for each interferogram. 

## 5. Discussion and Conclusions

Here, the spatial modulation depth and discrete phase are first obtained via the FT method, and the modulation threshold is then reasonably set to identify the pixels that are valid for phase unwrapping. After that, the intersection areas between two adjacent interferograms are utilized to conduct the phase stitching process. In this technique, the background noises and light fluctuations are substantially suppressed due to the signal process on each interferogram, and the moving errors are effectively eliminated because of the independent analysis of each interferogram. Importantly, the height range can be improved to multiple DOFs, which can be applied in various samples with heights from the nanometer level to the micrometer level. 

In conclusion, this paper has analyzed the theoretical principle, simulation and experimental validation of the phase stitching method for dimensional metrology of microstructures. The technique could achieve an improved robustness for the measurement of large-height microstructures, the advantages of which include the elimination of stepping errors, the suppression of light fluctuations, and the freedom of a limited DOF.

## Figures and Tables

**Table 1:** Experimental results using the proposed method and the 3D profiler.

Repetitions	(*x* = 300, *y* = 200)	(*x* = 400, *y* = 100)
	Height/nm	Height/nm
1	2639.2	2126.6
2	2638.1	2125.5
3	2637.9	2126.9
4	2639.9	2128.5
Average	2638.8	2126.9
3D Profiler	2640.2	2128.1
